# Development of a clinical prediction model using integrated epigenetics to predict COVID-19 severity

**DOI:** 10.1186/s40246-026-01017-7

**Published:** 2026-07-28

**Authors:** Cosby G. Arnold, James F. Holmes, Janine M. LaSalle, Daniel J. Tancredi, Iain R. Konigsberg, Ivana V. Yang, Michelle Edelmann, Monica Campbell, Kathleen C. Barnes, Andrew A. Monte

**Affiliations:** 1 Department of Emergency Medicine, University of California Davis Medical Center, Sacramento, USA; 2 Department of Medical Microbiology and Immunology, University of California, Davis, USA; 3 Department of Pediatrics, University of California Davis Medical Center, Sacramento, USA; 4 Department of Biomedical Informatics, University of Colorado Anschutz Medical Campus, Aurora, USA; 5 Department of Medicine, University of Colorado Anschutz Medical Campus, Aurora, USA; 6 University of Colorado Anschutz Medical Campus, Aurora, USA

**Keywords:** COVID-19, Methylation, Epigenetics, Biomarker, Clinical prediction model

## Abstract

**Background:**

Epigenetic markers, including DNA methylation (DNAm), can improve early disease diagnosis and risk stratification. The objective of this study was to evaluate whether the inclusion of a DNAm score would improve performance of a clinical prediction model for COVID-19 clinical severity.

**Methods:**

We performed a retrospective cohort study of adults (>18 years) with COVID-19 who presented to the emergency department from March to June 2020, the initial peak of the COVID-19 epidemic. DNAm scores were performed on a subset of the cohort based upon availability of blood specimens, to characterize epigenetic signatures. Clinical data were obtained from an electronic data warehouse that includes clinical and biological variables. The primary outcome was a modified WHO (mWHO) ordinal score for COVID-19 clinical improvement, which stratifies severity based on respiratory support required. We performed univariate and bivariate analyses to further select variables for inclusion (p<0.05). We used ordinal logistic regression to develop a model predicting mWHO score. DNAm scores were measured at presentation using modified Infinium MethylationEPIC Arrays with 7,831 additional probes targeting 262 immune-enriched genes. We assessed the DNAm score’s incremental impact on predictive model performance, using the estimated logit-hat for the model derived for the entire cohort as an independent variable in a pair of models fit to the DNAm subsample; one with and the other without the score as a variable. The Wald test was used to quantify the incremental predictive value of the DNAm score. Discriminative capacity was quantified and compared using the c-statistic transformation of Somers D.

**Results:**

A total of 1400 patients were included, 123 with DNAm scores. DNAm score had an adjusted odds ratio of 7.13 (95% CI 1.55-32.85; p=0.01). In comparing models with and without DNAm score, the c-statistic was 0.88 (95% CI 0.82-0.93; p<0.0001) without and 0.90 (95% CI 0.85-0.95; p<0.0001) with the DNAm score, an estimated difference in c-statistic of 0.02 (p=0.14).

**Conclusion:**

The addition of DNAm scores at presentation improved prediction but not model discrimination in this small sample.

**Supplementary Information:**

The online version contains supplementary material available at https://doi.org/10.1186/s40246-026-01017-7.

## Background

Acute respiratory tract infections pose a considerable threat to society. This was highlighted most recently by the emergence of severe acute respiratory syndrome coronavirus-2 (SARS-CoV-2), which rapidly evolved into a pandemic and overwhelmed the world’s healthcare systems [1]. In recent years, other emerging pathogens (i.e. influenza A virus subtype H1N1 or swine influenza, influenza A virus subtype H5N1 or avian influenza, Middle East respiratory syndrome [MERS]-CoV, and SARS-CoV), have caused epidemic and pandemic outbreaks responsible for severe pulmonary illness associated with high morbidity and mortality [2]. These viruses share a wide spectrum of severity, with most patients developing minor illness while some progress to respiratory failure and even death [3–6]. Identifying patients at highest risk for severe disease is critical for pandemic preparedness and response.

Epigenetic modifications, which refer to heritable alterations in gene expression without changes to the underlying deoxyribonucleic acid (DNA) sequence, modulate host immunity and inflammatory response to infection [7]. DNA methylation (DNAm) at cytosine-phosphate-guanine (CpG) sites, the most studied epigenetic modification, has been shown to predict cytokine levels [8]. However, unlike cytokines, which are challenging to measure [9], DNAm is biologically and technically stable [10], [11]. and potentially captures information about chronic comorbid conditions [12–14]. It is readily accessible in the peripheral blood which, although heterogeneous, is a reliable source for epigenetic signatures with well-documented utility for DNAm studies [15–18].

By revealing the underlying biology, epigenetic markers can expedite diagnosis and early identification of patients at high risk for severe disease. Enveloped RNA viruses, including coronaviruses, manipulate the host’s epigenome via DNAm [19–22]. These changes can differentiate SARS-CoV-2 infected from non-infected patients and identify patients who will progress to severe disease [23]. However, whether DNAmn patterns enhance the prognostic value of clinical prediction models remains unknown.

In this study, we used existing, detailed patient variables, alone and in combination with DNAm score, to predict disease severity and death among emergency department (ED) patients with COVID-19. We hypothesized that the inclusion of DNAm scores would enhance prediction. If successful, this would warrant the investigation of DNAm scores to predict outcomes in other severe disease processes.

## Methods

### Study design

The study protocol was approved by the Colorado Multiple Institutional Review Board (COMIRB) and the research adheres to the ethical principles outlined in the U.S. Federal Policy for the Protection of Human Subjects. All data were derived from the Health Data Compass Data Warehouse project (COMPASS www.healthdatacompass.org), a multi-institutional data warehouse that includes clinical and biological data from patients seen within the University of Colorado Health System (UCHealth). This retrospective analysis was specific to patients 18 years or older who met the following inclusion criteria: (1) SARS-CoV-2 + by RT-PCR or antigen test administered between March 1, 2020 and June 1, 2020; and (2) the test was performed within 48 h of an ED encounter. We excluded patients who died within 6 h of ED admission. Test results determined to be false positive, defined as a positive test followed by a negative test administered *≤* 24 h after the first test, were excluded. We used the earliest test as the index encounter in situations where individuals received multiple tests.

### Clinical outcome definition

We defined outcomes according to a modified version of the World Health Organization (mWHO) ordinal scale for clinical improvement, which stratifies COVID-19 severity based on level of respiratory support required [24]. We modified the score to ensure all scoring elements could be measured accurately with retrospective data capture. The 8 levels of the mWHO scale are as follows: (1) discharged from the ED without oxygen; (2) discharged from the ED with oxygen; (3) hospitalized, no oxygen therapy during admission; (4) hospitalized, oxygen required during admission; (5) hospitalized, required high-flow nasal cannula (HFNC) or non-invasive ventilation (NIV) during admission; (6) hospitalized, required invasive mechanical ventilation (IMV) during admission; (7) hospitalized, required IMV and additional organ support (e.g., extracorporeal membranous oxygen (ECMO), pressors); and (8) death. Any encounters within 30 days of an index encounter were assessed for the 8-class score and the maximum score, across all encounters, was used as the patient outcome for this analysis.

### Clinical variables

Data obtained from the electronic health record (EHR) included demographics, laboratory values, treatments, vital signs, and hospital disposition. Laboratory values and vital signs were captured for the first 6 h from the time of ED admission.

### DNAm score

DNAm scores were measured on first blood upon ED admission (hospital day 0); this typically occurs within 30 min of presentation. These scores were derived in a prior study with methods described in detail elsewhere [23]. Briefly, genomic DNA extracted from peripheral blood underwent bisulfite conversion prior to profiling on modified Infinium MethylationEPIC Arrays with 7831 additional probes targeting 262 immune-enriched genes. The SeSAMe R package was used for DNAm data processing and quality control, including normalization (noob), dye-bias correction, removal of probes with detection *p* > 0.05, probes overlapping common SNPs, poorly mapping probes, and probes containing non-unique sequences [25]. Beta values were transformed to M values for modeling. Penalized logistic regression (Lasso and Elastic Net) with tenfold cross-validation was used to develop the score in the parent study. The cohort used for the present analysis was selected from the prior study based on prespecified criteria. Therefore, we applied these previously derived DNAm scores to the present analysis without modification; we did not retrain or recalibrate the DNAm score model.

### Variable selection

We performed univariate and bivariate analyses on the study sample to assess variation and crude associations with the outcome, respectively. We included certain variables in the multivariate model based on prior literature and biologic plausibility. These variables included age, sex, initial vital signs (systolic and diastolic blood pressure, temperature, heart rate, and respiratory rate), body mass index (BMI), troponin, brain natriuretic peptide, D-dimer, lactate dehydrogenase (LDH), C-reactive protein (CRP), lactate, ferritin, glucose, creatinine, blood urea nitrogen (BUN), white blood cell count (WBC), neutrophils, lymphocytes, neutrophil to lymphocyte ratio, nucleated red blood cells (NRBC), granulocytes, and platelets [26–40]. We screened variables for collinearity prior to final selection. We used the missing indicator method for missing values [41]. This method creates a new binary indicator variable to indicate whether a given variable is missing. It is particularly useful for EHR data in which data is likely to be missing not at random, and in which the missingness pattern could provide information (i.e. perceived need for a test) that improves model predictive performance. The missing indicator approach has crucial advantages over multiple imputation for our application, in that it avoids the untestable and less tenable assumption of missingness at random while also being easier for others to implement or reproduce.

### Sample size

For this study, we gathered data on all patients in the study period. To indicate the precision of our estimates, we report all effect sizes with appropriate 95% confidence intervals [42].

### Model development and performance

To simplify our model at clinically meaningful thresholds, we dichotomized our outcome in two ways: WHO score < 2 versus *≥* 2; and WHO score < 4 versus *≥* 4. For the entire cohort, variables identified as significant and not colinear were entered into two separate binary logistic regression models, one for each of the dichotomized outcomes. We anticipated that if the selected features and logistic regression coefficients were similar for these two models, we would develop an ordinal three-level outcome (WHO score < 2 versus WHO score equal to either 2 or 3 versus WHO score *≥* 4) and model it using ordinal logistic regression.

To take best advantage of study data in controlling for baseline covariates, we used the logistic regression coefficients for the predictive model estimated using the entire cohort to derive logit-hats for individuals in the much smaller DNAm subsample that could then be used as an independent variable in the pair of models that were used to evaluate the incremental value of DNAm score for that subsample, with one model including and one model excluding the DNAm score as an additional independent variable. The statistical significance of the Wald test for the regression coefficient for the DNAm score in the former model was used for testing the predictive value of the methylation score [43], whereas the added discriminative capacity was quantified by comparing the area under the curve statistics for the two models, using the somersd package in Stata with the Harrell C-transformation applied to the Somers D ( prediction | outcome ), C = 0.5*(1 + D), where C is the area under the curve and D is Somers D, a transformation that is applicable to an ordinal outcome with 2 or more levels [44]. We also used this package and transformation to quantify the discriminative capacity of the predictive model developed for the full cohort.

Finally, we considered that the DNAm score could be acting as a surrogate for medical comorbidities (the original epigenome-wide association study adjusted for age and sex but not comorbidities). Thus, we refit the model with the Elixhauser Comorbidity Index [45]. to evaluate change in overall model performance and significance of the DNAm score.

### Enrichment analysis

We performed gene set enrichment analysis of differentially methylated positions using the missMethyl R package [46], which accounts for bias due to differing numbers of probes per gene. Enrichment was evaluated using Gene Ontology (GO) categories and Kyoto Encyclopedia of Genes and Genomes (KEGG) pathways to map DNAm changes to biological processes and pathways, as previously described [23].

## Results

### Selection of the study cohort

A total of 41,128 COVID-19 PCR or antigen tests were administered during the study period. 3,601 (8.8%) tests were positive and, of these, sixteen (0.4%) tests were determined to be false positive and removed from the analysis. The remaining 3,585 tests were attributed to 2,907 individuals indicating that some had multiple tests.

**Fig. 1:**
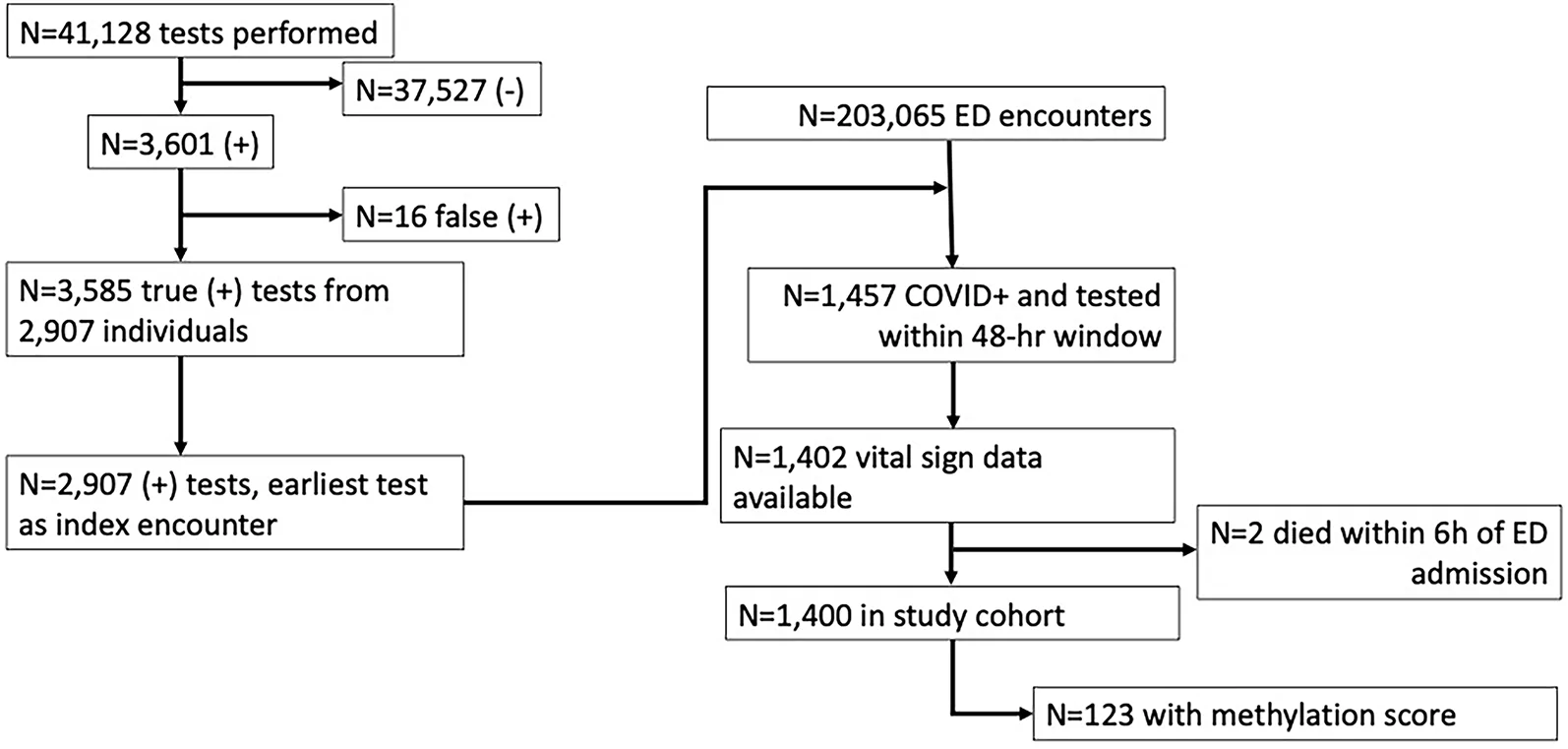
Study cohort selection

The COMPASS dataset contained 203,065 ED encounters, of which 1457 occurred within the 48-hour window of COVID-19 testing. A total of 1,402 (96%) of the 1457 had vital sign data in COMPASS within the first 6 h of ED admission. Two of these patients died within 6 h of ED admission and were excluded, leaving 1400 patients for our analysis. All 164 patients with DNAm scores were re-identified in COMPASS and, of these, 123 met our eligibility criteria and were included in our subanalysis (Fig. 1).

### Characteristics of the study cohort

Of the 1,400 patients in our cohort, 784 (56%) were male and the mean age was 53.9 years (standard deviation 17.9 years). Baseline characteristics for the study cohort are presented in Table 1.

**Table 1:** Demographic and clinical characteristics by WHO severity score category

	WHO severity score category			
	WHO 1 (*N* = 322)	WHO 2 or 3 (*N* = 112)	WHO *≥* 4 (*N* = 966)	Total (*N* = 1400)
Age(years)				
Mean (SD)	43.1 (13.85)	49.0 (19.29)	58.1 (17.28)	53.9 (17.90)
Sex, n (%)				
Female	169 (52.5%)	53 (47.3%)	394 (40.8%)	616 (44.0%)
Race/Ethnicity, n (%)				
Hispanic/Latino White, Non-Hispanic Black, Non-Hispanic Other	176 (54.7%) 55 (17.1%) 44 (13.7%) 47 (14.6%)	58 (51.8%) 26 (23.2%) 18 (16.1%) 10 (8.9%)	418 (43.3%) 307 (31.8%) 137 (14.2%) 104 (10.8%)	652 (46.6%) 388 (27.7%) 199 (14.2%) 161 (11.5%)
BMI				
Mean (SD)	30.2 (7.01)	29.2 (6.48)	31.6 (7.87)	31.2 (7.70)
Blood Glucose (mg/dL)				
Mean (SD)	125.7 (61.99)	144.2 (116.61)	152.5 (91.88)	148.1 (91.31)
Blood urea nitrogen (BUN) (mg/dL)				
Mean (SD)	12.8 (5.60)	15.7 (10.52)	21.5 (20.95)	19.8 (19.10)
NLR				
Mean (SD)	3.6 (3.30)	4.2 (3.56)	6.6 (5.96)	6.0 (5.62)
Diastolic blood pressure				
Mean (SD)	81.7 (13.72)	81.5 (14.33)	76.8 (14.79)	78.3 (14.68)
Heart rate				
Mean (SD)	95.5 (17.72)	97.6 (19.57)	99.7 (19.21)	98.6 (18.97)
Respiratory rate				
Mean (SD)	18.8 (3.43)	19.6 (3.71)	22.0 (6.03)	21.1 (5.55)
Temperature,** F**				
Mean (SD)	98.6 (1.41)	98.4 (1.25)	99.1 (1.68)	98.9 (1.61)
Methylation score				
Mean (SD)	1.8 (0.29)	1.8 (0.28)	2.2 (0.44)	2.1 (0.44)

*SD* standard deviation, *BMI* body mass index, *NLR* neutrophil: lymphocyte ratio, *F* Fahrenheit

### Variable selection

Informed by univariate and bivariate analyses, we retained 9 features for our multivariable regression modeling: sex, age, BMI, glucose, neutrophil: lymphocyte ratio (NLR), BUN, earliest diastolic blood pressure, earliest heart rate, and earliest temperature. To avoid excluding patients with missing values from the analysis, we added additional levels to categorical values to indicate missingness for the following variables: BMI (18.5–29.9, < 18.5 or > 30, missing), glucose (71–119, < 70 or > 120, missing), BUN (< 20, > 20, missing), and NLR (0.7-3.0, < 0.7 or > 3.0, missing).

### Model development and performance

Our ordinal regression model derived for the entire cohort of 1,400 patients resulted in a c-statistic of 0.88 (95% CI 0.82–0.93; *p* < 0.0001). See Table 2 for odds ratios for model covariates.

**Table 2:** Model covariates in 3-level ordinal regression model for WHO severity score

	Odds ratio estimates and wald confidence intervals		
	OR	95% CI	
Sex Male vs. Female	1.81	1.34	2.46
Age 18–44 years vs. Age 45–64 years	0.42	0.3	0.59
Age *≥* 65 years vs. Age 45–64 years	3.88	2.44	6.16
Unhealthy BMI (< 18.5 or *≥* 30) vs. Healthy BMI	2.16	1.5	3.12
Abnormal glucose (*≤* 70 or *≥* 120) vs. Normal glucose	1.75	1.25	2.43
Abnormal NLR (< 0.7 or > 3.0) vs. Normal NLR	1.92	1.34	2.76
Diastolic blood pressure (per 10-unit increase)	0.85	0.77	0.95
Heart rate	1.02	1.01	1.03
Respiratory rate	1.09	1.05	1.13
Temperature	1.06	0.96	1.17
Methylation score	7.13	1.53	33.18

*BMI* body mass index, *NLR* neutrophil: lymphocyte ratio

A subsample of 123 patients had DNAm scores. For this subsample, we used the estimated logit-hat from the model fit the entire cohort as an independent variable in a pair of additional models; one model included the DNAm score as an additional independent variable. The full ordinal model is provided in Supplementary Table 1. The DNAm score had an adjusted odds ratio of 7.13 (95% CI 1.55–32.85; *p* = 0.01). Addition of the DNAm score to the ordinal regression model increased the c-statistic from 0.88 (95% CI 0.82–0.93; *p* < 0.0001) to 0.90 (95% CI 0.85–0.95; *p* < 0.0001), with an estimated difference in c-statistic of 0.02 (*p* = 0.14). The proportional-odds assumption was supported by the Brant test (*p* = 0.27).

Our model refit with the Elixhauser Comorbidity Index had an adjusted odds ratio of 6.77 (95% CI 1.24–37.10; *p* = 0.03). The c-statistic for the ordinal regression model without DNAm score was 0.89 (95% CI 0.84–0.95; *p* < 0.0001). The c-statistic for the ordinal regression model with DNAm score was 0.91 (95% CI 0.86–0.95; *p* < 0.0001). The difference in c-statistics was 0.02 (*p* = 0.25).

### Enrichment analysis

**Fig. 2:**
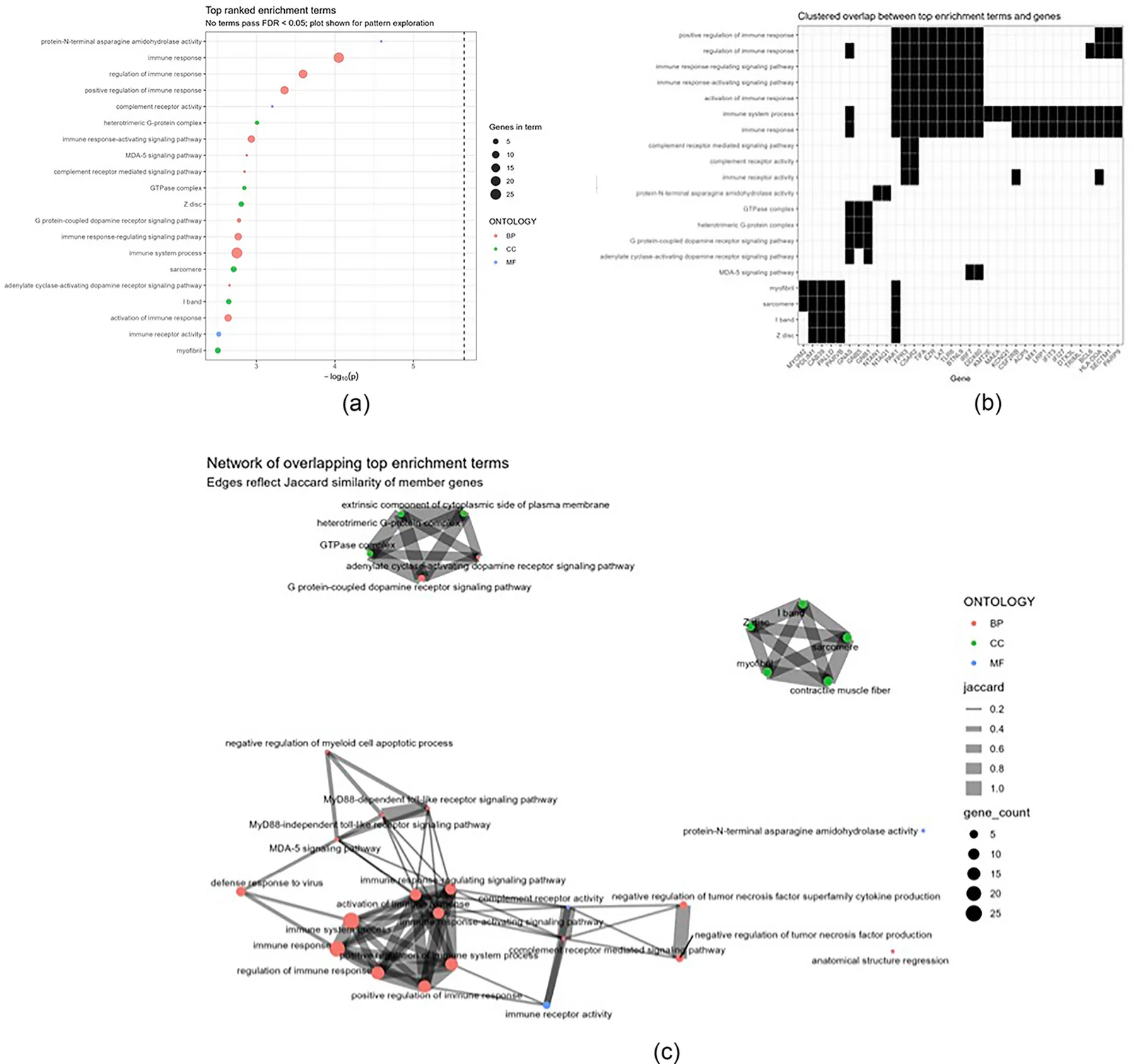
**a** Enrichment plot shows top ranked enrichment terms in immune-related pathways. These did not reach FDR significance in our small sample size.** b** Heatmap demonstrates clustering of genes and top pathways.** c** Network plot explores enriched terms based on gene overlap. * BP* biological process;* CC* cellular component;* MF* Molecular function

Top enrichment terms from DNAm pathway analysis were predominantly related to immune response pathways; however, these associations did not reach statistical significance (Fig. 2). This is likely due to the limited number of CpGs included in the DNAm score, which reduces power for pathway-level enrichment analyses, in contrast to our prior work demonstrating significant enrichment for interferon and antiviral response pathways [23].

## Discussion

In this retrospective cohort study, we used EHR and DNAm data to predict disease severity among ED patients with COVID-19. We developed an ordinal regression model that performed with high discriminative capacity. Addition of the DNAm score improved model performance, but the improvement in discriminative capacity was not statistically significant.

An ideal model would rapidly discern between those patients who develop severe clinical outcomes and those who do not. Both our models had excellent discriminative capacity (c-statistics > 0.80). Our initial model, limited to routinely available clinical variables, had a c-statistic of 0.88. Addition of DNAm score further increased the c-statistic to 0.90. This change did not reach statistical significance, likely due to the small cohort size of the methylation subsample. However, that inclusion of the DNAm score improved the c-statistic, which was already quite high, suggests that DNAm score provides additional, important information not fully captured by the other model variables.

We did not include the Elixhauser Comorbidity Index in our initial model because it is cumbersome and not used routinely in most settings. However, because DNAm has been shown to capture information about chronic comorbid conditions [12–14]. we refit our model with the Elixhauser Comorbidity Index included as an additional covariate. We found that the DNAm score adjusted odds ratio decreased from 7.13 to 6.77, remaining an important predictor and capturing information not included in Elixhauser Comorbidity Index.

Numerous prognostic tools have been developed for COVID-19. Haimovich et al. [47]. developed the quick COVID-19 severity index to predict respiratory failure within 24 h of admission in adults with COVID-19 requiring supplemental oxygen (c-statistic 0.81; 95% CI 0.73–0.89). Liang et al. [48]. developed a clinical risk score to predict critical illness, defined as intensive care unit admission, invasive mechanical ventilation, or death, based on 10 variables measured at the time of admission to the hospital (c-statistic 0.88–0.91). Goodacre et al. [49]. reported a c-statistic of 0.80 (95% CI 0.79–0.81) for a clinical score based on NEWS2, age, sex, and performance status to predict death or major organ support within 30 days after initial presentation to the ED. These studies were limited to patients requiring hospitalization or critical illness support whereas the current study included all ED admissions.

Novel biomarkers, including those derived from multiplexed ‘omics technologies, can improve diagnosis and risk stratification, as has been demonstrated in sepsis, hemorrhagic shock after trauma, and cancer [50–52]. Our findings suggest a potential role for integrating epigenetic markers with clinical data to improve prediction of clinical outcomes in COVID-19 and perhaps other respiratory diseases. However, the results of our study require further evaluation, to include prospective validation, external validation, and assessment of clinical utility prior to considering clinical implementation.

Although DNAm data is not routinely available in the ED, there is reason for optimism that integrating epigenetics into clinical-decision making will improve clinical outcomes and be cost-saving in the future. For instance, Illumina short-read sequencing costs have decreased > 1000-fold over the past 20 years and targeted or whole genome bisulfite sequencing are becoming less expensive than array-based approaches [53]. Long-read sequencing by Oxford Nanopore Technologies can directly read DNAm data from targeted regions in real time, enabling fast decisions about clinical care, as in the case of brain tumors [54, 55]. Furthermore, epigenetic biomarker use can potentially save costs by facilitating early interventions to minimize clinical severity of illness and by preserving resources in determining which patients do not need hospitalization or significant follow-up. For example, particularly early in the COVID-19 pandemic, rapid identification of patients at highest risk for severe disease would prioritize distribution of treatments such as remdesivir [56]. or dexamethasone [57].

This study is important not just for COVID-19 but also for other critical conditions in which there is prognostic uncertainty at presentation. An individual’s methylome varies acutely based on the clinical condition or environmental exposure, providing additional insight into the individual’s pathobiology. With integrative epigenetics, clinicians would have additional knowledge of anticipated clinical outcomes and higher confidence in their clinical decision-making.

## Limitations

Our study has limitations. First, COMPASS includes only patient data from UCHealth. However, this healthcare system encompasses a large, integrated network of clinics and hospitals that, when combined, includes more than half of Coloradoans. Second, this is a retrospective study and subject to the inherent limitations of this design. Third, our study was restricted to the first three months of the COVID-19 pandemic, prior to vaccines being available and with limited treatment options. The disease has become less severe as it has evolved and therefore, both the pertinent methylome and the clinical variables may have shifted from when these data were collected. However, the methodology may be instructive for other conditions that are less temporally variable in severity. Fourth, the number of patients with DNAm data available was relatively small. Finally, our findings cannot fully differentiate intrinsic epigenetic regulation from changes in immune-cell composition. Blood-derived DNAm profiles may capture both disease-associated epigenetic differences and acute changes in circulating immune-cell composition. In the parent study [23], epigenome-wide analyses incorporated ReFACTor to account for cell-type heterogeneity, and the identified methylation signature was not explained by peripheral blood cell proportions measured from complete blood counts when evaluated for potential confounding. Future studies using more comprehensive cell-type deconvolution methods or purified cell populations could further clarify the biological mechanisms underlying the DNAm score. Nevertheless, our findings are still relevant in demonstrating the utility of integrating epigenetics to enhance the predictive ability of routinely available clinical variables and to optimize resource utilization.

## Conclusions

We derived and internally validated a model to predict COVID-19 severity in adult ED patients. Inclusion of DNAm score improved model performance, although the improvement in discriminative capacity was not significant. Although this study was limited by the small number of DNAm scores available, it provides a proof of concept for the utility of integrated epigenetics in prediction modeling. Future studies that employ larger data sets and associated biobanks should be considered to further support the value of these models.

## Supplementary Information

Supplementary Material 1.

## Data Availability

Availability of data and materials: Data is available upon request.

## References

[1] Li Q, Guan X, Wu P, et al. Early Transmission Dynamics in Wuhan, China, of Novel Coronavirus-Infected Pneumonia. N Engl J Med. 2020;382(13):1199–207. 10.1056/NEJMoa2001316. PMC7121484 31995857

[2] Gillim-Ross L, Subbarao K. Emerging respiratory viruses: challenges and vaccine strategies. Clin Microbiol Rev. 2006;19(4):614–36. 10.1128/cmr.00005-06. PMC1592697 17041137

[3] Nicholls JM, Poon LL, Lee KC, et al. Lung pathology of fatal severe acute respiratory syndrome. Lancet. 2003;361(9371):1773–8. 10.1016/s0140-6736(03)13413-7. PMC7112492 12781536

[4] Ding Y, He L, Zhang Q, et al. Organ distribution of severe acute respiratory syndrome (SARS) associated coronavirus (SARS-CoV) in SARS patients: implications for pathogenesis and virus transmission pathways. J Pathol. 2004;203(2):622–30. 10.1002/path.1560. PMC7167761 15141376

[5] Bradley BT, Bryan A. Emerging respiratory infections: The infectious disease pathology of SARS, MERS, pandemic influenza, and Legionella. Semin Diagn Pathol. 2019;36(3):152–9. 10.1053/j.semdp.2019.04.006. PMC7125557 31054790

[6] Gupta S, Hayek SS, Wang W, et al. Factors associated with death in critically ill patients with Coronavirus Disease 2019 in the US. JAMA Intern Med. 2020;180:1–12. 10.1001/jamainternmed.2020.3596. PMC7364338 32667668

[7] Busslinger M, Tarakhovsky A. Epigenetic control of immunity. Cold Spring Harb Perspect Biol. 2014;6(6). 10.1101/cshperspect.a019307. PMC4031963 24890513

[8] Lorente-Sorolla C, Garcia-Gomez A, Català-Moll F, et al. Inflammatory cytokines and organ dysfunction associate with the aberrant DNA methylome of monocytes in sepsis. Genome Med. 2019;11(1):66. 10.1186/s13073-019-0674-2. PMC6820973 31665078

[9] Liu C, Chu D, Kalantar-Zadeh K, George J, Young HA, Liu G. Cytokines: From Clinical Significance to Quantification. Adv Sci (Weinh). 2021;8(15):e2004433. 10.1002/advs.202004433. PMC8336501 34114369

[10] Gosselt HR, Griffioen PH, van Zelst BD, Oosterom N, de Jonge R, Heil SG. Global DNA (hydroxy)methylation is stable over time under several storage conditions and temperatures. Epigenetics. 2021;16(1):45–53. 10.1080/15592294.2020.1786318. PMC7889142 32614650

[11] Pidsley R, Zotenko E, Peters TJ, et al. Critical evaluation of the Illumina MethylationEPIC BeadChip microarray for whole-genome DNA methylation profiling. Genome Biol. 2016;17(1):208. 10.1186/s13059-016-1066-1. (In eng). PMC5055731 27717381

[12] Jin Z, Liu Y. DNA methylation in human diseases. Genes Dis. 2018;5(1):1–8. 10.1016/j.gendis.2018.01.002. PMC6147084 30258928

[13] Stylianou E. Epigenetics of chronic inflammatory diseases. J Inflamm Res. 2019;12:1–14. 10.2147/jir.S129027. PMC6304253 30588059

[14] Wielscher M, Mandaviya PR, Kuehnel B, et al. DNA methylation signature of chronic low-grade inflammation and its role in cardio-respiratory diseases. Nat Commun. 2022;13(1):2408. 10.1038/s41467-022-29792-6. PMC9065016 35504910

[15] Beltrán-García J, Casabó-Vallés G, Osca-Verdegal R, et al. Alterations in leukocyte DNA methylome are associated to immunosuppression in severe clinical phenotypes of septic patients. Front Immunol. 2023;14:1333705. 10.3389/fimmu.2023.1333705. PMC10791922 38235139

[16] Bos LDJ, Scicluna BP, Ong DSY, Cremer O, van der Poll T, Schultz MJ. Understanding Heterogeneity in Biologic Phenotypes of Acute Respiratory Distress Syndrome by Leukocyte Expression Profiles. Am J Respir Crit Care Med. 2019;200(1):42–50. 10.1164/rccm.201809-1808OC. (In eng). 30645145

[17] Panagopoulou M, Karaglani M, Balgkouranidou I, et al. Circulating cell-free DNA in breast cancer: size profiling, levels, and methylation patterns lead to prognostic and predictive classifiers. Oncogene. 2019;38(18):3387–401. 10.1038/s41388-018-0660-y. 30643192

[18] Wang C, Chen L, Yang Y, Zhang M, Wong G. Identification of potential blood biomarkers for Parkinson’s disease by gene expression and DNA methylation data integration analysis. Clin Epigenetics. 2019;11(1):24. 10.1186/s13148-019-0621-5. PMC6371578 30744671

[19] Menachery VD, Eisfeld AJ, Schäfer A, et al. Pathogenic influenza viruses and coronaviruses utilize similar and contrasting approaches to control interferon-stimulated gene responses. mBio. 2014;5(3):e01174–14. 10.1128/mBio.01174-14. PMC4030454 24846384

[20] Menachery VD, Schäfer A, Burnum-Johnson KE, et al. MERS-CoV and H5N1 influenza virus antagonize antigen presentation by altering the epigenetic landscape. Proc Natl Acad Sci USA. 2018;115(5):E1012–21. 10.1073/pnas.1706928115. PMC5798318 29339515

[21] Schäfer A, Baric RS. Epigenetic Landscape during Coronavirus Infection. Pathogens. 2017;6(1). 10.3390/pathogens6010008. PMC5371896 28212305

[22] Totura AL, Baric RS. SARS coronavirus pathogenesis: host innate immune responses and viral antagonism of interferon. Curr Opin Virol. 2012;2(3):264–75. 10.1016/j.coviro.2012.04.004. PMC7102726 22572391

[23] Konigsberg IR, Barnes B, Campbell M, et al. Host methylation predicts SARS-CoV-2 infection and clinical outcome. Comms Med. 2021;1(42):1–10. 10.1038/s43856-021-00042-y PMC8767772 35072167

[24] A minimal common outcome measure set for COVID-19 clinical research. Lancet Infect Dis. 2020;20(8):e192–7. 10.1016/s1473-3099(20)30483-7. PMC7292605 32539990

[25] Zhou W, Triche TJ Jr., Laird PW, Shen H. SeSAMe: reducing artifactual detection of DNA methylation by Infinium BeadChips in genomic deletions. Nucleic Acids Res. 2018;46(20):e123. 10.1093/nar/gky691. PMC6237738 30085201

[26] Barrett TJ, Bilaloglu S, Cornwell M, et al. Platelets contribute to disease severity in COVID-19. J Thromb haemostasis: JTH. 2021;19(12):3139–53. 10.1111/jth.15534. (In eng). PMC8646651 34538015

[27] Cihakova D, Streiff MB, Menez SP et al. High-value laboratory testing for hospitalized COVID-19 patients: a review. Future Virol 2021 (In eng). 10.2217/fvl-2020-0316 PMC8457535 34567235

[28] Düz ME, Arslan M, Menek EE, Avci BY. Impact of the seventh day nucleated red blood cell count on mortality in COVID-19 intensive care unit patients: A retrospective case-control study. J Med Biochem. 2023;42(1):138–44. 10.5937/jomb0-39839. (In eng). PMC9920868 36819135

[29] Georgakopoulou VE, Makrodimitri S, Triantafyllou M, et al. Immature granulocytes: Innovative biomarker for SARS–CoV–2 infection. Mol Med Rep. 2022;26(1). 10.3892/mmr.2022.12733. (In eng). PMC9175277 35551416

[30] Ouyang SM, Zhu HQ, Xie YN, et al. Temporal changes in laboratory markers of survivors and non-survivors of adult inpatients with COVID-19. BMC Infect Dis. 2020;20(1):952. 10.1186/s12879-020-05678-0. (In eng). PMC7729703 33308159

[31] Sarkar S, Khanna P, Singh AK. The Impact of Neutrophil-Lymphocyte Count Ratio in COVID-19: A Systematic Review and Meta-Analysis. J Intensive Care Med. 2022;37(7):857–69. 10.1177/08850666211045626. (In eng). PMC9160638 34672824

[32] Terpos E, Ntanasis-Stathopoulos I, Elalamy I, et al. Hematological findings and complications of COVID-19. Am J Hematol. 2020;95(7):834–47. 10.1002/ajh.25829. (In eng). PMC7262337 32282949

[33] Wool GD, Miller JL. The Impact of COVID-19 Disease on Platelets and Coagulation. Pathobiology. 2021;88(1):15–27. 10.1159/000512007. (In eng). PMC7649697 33049751

[34] Yang X, Jin Y, Li R, Zhang Z, Sun R, Chen D. Prevalence and impact of acute renal impairment on COVID-19: a systematic review and meta-analysis. Crit Care. 2020;24(1):356. 10.1186/s13054-020-03065-4. (In eng). PMC7300374 32552872

[35] Zahedi M, Kordrostami S, Kalantarhormozi M, Bagheri M. A Review of Hyperglycemia in COVID-19. Cureus. 2023;15(4):e37487. 10.7759/cureus.37487. PMC10181889 37187644

[36] Zahorec R, Hulin I, Zahorec P. Rationale Use of Neutrophil-to-lymphocyte ratio for early diagnosis and stratification of COVID-19. Bratisl Lek Listy. 2020;121(7):466–70. 10.4149/bll_2020_077. (In eng). 32989997

[37] Zinellu A, Mangoni AA. A systematic review and meta-analysis of the association between the neutrophil, lymphocyte, and platelet count, neutrophil-to-lymphocyte ratio, and platelet-to-lymphocyte ratio and COVID-19 progression and mortality. Expert Rev Clin Immunol. 2022;18(11):1187–202. 10.1080/1744666x.2022.2120472. (In eng). 36047369

[38] Alwani M, Yassin A, Al-Zoubi RM, et al. Sex-based differences in severity and mortality in COVID-19. Rev Med Virol. 2021;31(6):e2223. 10.1002/rmv.2223. PMC8014761 33646622

[39] Du Y, Lv Y, Zha W, Zhou N, Hong X. Association of body mass index (BMI) with critical COVID-19 and in-hospital mortality: A dose-response meta-analysis. Metabolism. 2021;117:154373. 10.1016/j.metabol.2020.154373. PMC7493748 32949592

[40] Hu B, Guo H, Zhou P, Shi ZL. Characteristics of SARS-CoV-2 and COVID-19. Nat Rev Microbiol. 2021;19(3):141–54. 10.1038/s41579-020-00459-7. PMC7537588 33024307

[41] Sperrin M, Martin GP, Sisk R, Peek N. Missing data should be handled differently for prediction than for description or causal explanation. J Clin Epidemiol. 2020;125:183–7. 10.1016/j.jclinepi.2020.03.028. 32540389

[42] Greenland S, Senn SJ, Rothman KJ, et al. Statistical tests, P values, confidence intervals, and power: a guide to misinterpretations. Eur J Epidemiol. 2016;31(4):337–50. 10.1007/s10654-016-0149-3. PMC4877414 27209009

[43] Vickers AJ, Cronin AM, Begg CB. One statistical test is sufficient for assessing new predictive markers. BMC Med Res Methodol. 2011;11:13. 10.1186/1471-2288-11-13. PMC3042425 21276237

[44] Newson R, SOMERSD. Stata module to calculate Kendall’s tau-a, Somers’ D and median differences. Statistical Software Components S336401 2020(revised 16 Apr 2020):Boston College Department of Economics.

[45] Elixhauser A, Steiner C, Harris DR, Coffey RM. Comorbidity measures for use with administrative data. Med Care. 1998;36(1):8–27. 10.1097/00005650-199801000-00004. 9431328

[46] Phipson B, Maksimovic J, Oshlack A. missMethyl: an R package for analyzing data from Illumina’s HumanMethylation450 platform. Bioinformatics. 2016;32(2):286–8. 10.1093/bioinformatics/btv560. 26424855

[47] Haimovich AD, Ravindra NG, Stoytchev S, et al. Development and validation of the Quick COVID-19 Severity Index: a prognostic tool for early clinical decompensation. Ann Emerg Med. 2020;76(4):442–53. 10.1016/j.annemergmed.2020.07.022. PMC7373004 33012378

[48] Liang W, Liang H, Ou L, et al. Development and Validation of a Clinical Risk Score to Predict the Occurrence of Critical Illness in Hospitalized Patients With COVID-19. JAMA Intern Med. 2020;180(8):1081–9. 10.1001/jamainternmed.2020.2033. PMC7218676 32396163

[49] Goodacre S, Thomas B, Sutton L, et al. Derivation and validation of a clinical severity score for acutely ill adults with suspected COVID-19: The PRIEST observational cohort study. PLoS ONE. 2021;16(1):e0245840. 10.1371/journal.pone.0245840. PMC7822515 33481930

[50] D’Alessandro A, Nemkov T, Moore HB, et al. Metabolomics of trauma-associated death: shared and fluid-specific features of human plasma vs lymph. Blood Transfus. 2016;14(2):185–94. 10.2450/2016.0208-15. PMC4918547 27177401

[51] Nemkov T, D’Alessandro A, Reisz JA. Metabolic underpinnings of leukemia pathology and treatment. Cancer Rep (Hoboken). 2019;2(2):e1139. 10.1002/cnr2.1139. PMC7941580 32721091

[52] Wong HR, Lindsell CJ, Pettilä V, et al. A multibiomarker-based outcome risk stratification model for adult septic shock. Crit Care Med. 2014;42(4):781–9. 10.1097/ccm.0000000000000106. PMC4620515 24335447

[53] Suzuki M, Liao W, Wos F, et al. Whole-genome bisulfite sequencing with improved accuracy and cost. Genome Res. 2018;28(9):1364–71. 10.1101/gr.232587.117. PMC6120621 30093547

[54] Shelton WJ, Zandpazandi S, Nix JS, et al. Long-read sequencing for brain tumors. Front Oncol. 2024;14:1395985. 10.3389/fonc.2024.1395985. PMC11194609 38915364

[55] Vermeulen C, Pagès-Gallego M, Kester L, et al. Ultra-fast deep-learned CNS tumour classification during surgery. Nature. 2023;622(7984):842–9. 10.1038/s41586-023-06615-2. PMC10600004 37821699

[56] Beigel JH, Tomashek KM, Dodd LE, et al. Remdesivir for the treatment of COVID-19 - final report. N Engl J Med. 2020;383:1813–26. 10.1056/NEJMoa2007764. PMC7262788 32445440

[57] Horby P, Lim WS, Emberson JR et al. Dexamethasone in hospitalized patients with COVID-19 - preliminary report. N Engl J Med. 2020. 10.1056/NEJMoa2021436 PMC7383595 32678530

