# Supplementary material for: Development of a clinical prediction model using integrated epigenetics to predict COVID-19 severity
Source: Hum Genomics. 2026 Jul 28;20:148. doi: 10.1186/s40246-026-01017-7 (PMC13711944; doi:10.1186/s40246-026-01017-7)
Supplement: Supplementary file 1 [file 40246_2026_1017_MOESM1_ESM.docx]

**Supplementary Table 1. Parameter estimates and Wald confidence intervals**

| **Parameter** |  | **Estimate** | **95% Confidence intervals** | |
| --- | --- | --- | --- | --- |
| Intercept | 2 | -4.2363 | -7.2533 | -1.2193 |
| Intercept | 1 | -3.2532 | -6.2104 | -0.2961 |
| Logit-hat |  | 1.0599 | 0.6467 | 1.4731 |
| Methylation score |  | 1.9274 | 0.4029 | 3.4519 |
